# Editorial: Polyphenols' action on the brain

**DOI:** 10.3389/fnins.2022.947761

**Published:** 2022-07-21

**Authors:** Eglantine Balland, Pauline Lafenetre, David Vauzour

**Affiliations:** ^1^Department of Nutrition, Dietetics and Food, School of Clinical Sciences at Monash Health, Faculty of Medicine, Nursing and Health Sciences, Monash University, Notting Hill, VIC, Australia; ^2^Bordeaux INP, Nutrition and Integrative Neurobiology, UMR1286, Bordeaux, France; ^3^CNRS, Interdisciplinary Institute for Neuroscience, UMR 5297, Bordeaux, France; ^4^Norwich Medical School, Biomedical Research Centre, Faculty of Medicine and Health Sciences, University of East Anglia, Norwich, United Kingdom

**Keywords:** neuroprotection, nutrition, polyphenol, neurodegenerative disease, blood-brain barrier, antioxidants, brain health

Unlike humans, plants do not possess an immune system to protect them from biological, physical and chemical threats, hence they had to develop different strategies to protect themselves from predation and other environmental menaces. (Poly)phenols are secondary plant metabolites able to fulfill this protective role, so why wouldn't they also confer health benefits to humans? Over the last decades, a great body of scientific evidence has demonstrated the diverse health benefits exerted by these bioactive compounds on human physiology. (Poly)phenols represent a fantastic preventive treatment opportunity against many pathologies, including neurodegenerative diseases, as they are already present in many plant-based foods we consume, contributing to their color, flavor, bitterness, astringency and oxidative stability, and therefore are safe to prescribe. Phenolic acids and flavonoids are the (poly)phenols the most abundantly found in plant-based food consumed by humans.

Since (poly)phenols are known to cross (Figueira et al., 2017) and possibly modulate (Lee et al., 2020), the blood-brain barrier (BBB) and have been shown to play a protective role in neurodegenerative diseases such as Parkinson's and Alzheimer's diseases (Ramassamy, 2006), this Research Topic focuses on the health benefits exerted by (poly)phenols specifically on the brain. Studying the molecular mechanisms of action of (poly)phenols on human physiology represents a challenge for various reasons. First, (poly)phenols can have some profound effects *in vitro*, when a particular metabolite is applied at high dose. However, in many cases such high dose isn't achievable with normal food consumption and the bioactivity and bioavailability will differ greatly when used *in vivo*. Various metabolites with different potency and actions can be formed according to where (which organ or cell type) they are metabolized. Additionally, (poly)phenols can also be extensively transformed by the gut microbiota and results in numerous novel compounds harboring a range of different physiological effects. It is therefore important to take these limitations into account and to use multi-dimensional approaches along with various models (*in vitro*, animal models, and human studies) when investigating the health benefits of (poly)phenols, and in this particular context, be aware of 1/ the possibility of a specific polyphenol to access the central nervous system (CNS) and 2/ knowing which metabolite(s) derived from the polyphenol of interest is/are likely to act on the brain microenvironment and target cells.

This Research Topic of articles gathers studies demonstrating various health benefits exerted by (poly)phenols on the brain. Experimental results obtained from cell lines, rodents, and human studies measure (poly)phenols' health benefits at the molecular level and show how these mechanisms may translate into behavioral effects.

Accuracy and speed of attention analyzes represent a good measurement of some of the function of a healthy brain. In their meta-analysis of the results from 18 of the most recent randomized controlled trials, Hepsomali et al. highlighted the significant positive effect of (poly)phenol consumption (various compounds and doses) on accuracy and speed of attention, independently of age and gender. Pontifex et al. studied more specifically the effect of citrus fruits-derived (poly)phenols on various homeostatic and physio-pathologic events occurring in/affecting the CNS such as depression, schizophrenia, stroke, dementia, cellular processes contributing to the maintenance of homeostasis, gut/brain cross-talk, antioxidant, and anti-inflammatory activities. The authors analyzed and summarized the results from *in vitro* and pre-clinical studies, using isolated compounds or whole fruit or mixture of compounds in a juice, as well as molecular and behavioral studies performed in rodents with dietary supplementation or injections of citrus-derived polyphenols to demonstrate that hesperidin, hesperetin, naringenin, naringin, and kaempferol consistently exert beneficial effects on the CNS-dependent parameters studied.

Tg4510 is a mutant mouse model of Alzheimer's disease which spontaneously develops TAU pathology, one of the hallmarks of the neurodegenerative disease in which the hyperphosphorylation of TAU protein leads to its aggregation and propagation, contributing to neuronal dysfunction and ultimately neuronal death. The oral administration of relatively high dose of the flavonoid epicatechin for 3 weeks in older mutant mice was able to prevent the development of TAU pathology in this murine model (Hole et al.). While the molecular mechanisms of this protective effect remain to be further investigated, it would be interesting to see whether this preventive effect translates into better cognitive outcomes as well.

Epicatechin has also known beneficial effects on cognitive functions and has the potential to prevent neurodegenerative disorders. Corral-Jara et al. explored a possible molecular mechanism underlying the neuroprotective actions of this flavanol, at the level of the vasculature composing the anatomical and physiological barrier between the blood and the brain. To replicate the physiopathological conditions of neurodegenerative diseases, inflammatory stress was mimicked using TNF-a in the culture medium of brain endothelial cells. After treatment of the cells with 5-(4'-Hydroxyphenyl)-γ-valerolactone-3'-sulfate and 5-(4'-Hydroxyphenyl)-γ-valerolactone-3'-O-glucuronide, the two major products of gut microbiota catabolism of epicatechin, the authors investigated the multi-omics responses. Study of gene expression, micro-RNAs, non-coding RNAs and proteins revealed that microbiome-derived metabolites of epicatechin modulate the expression of genes and proteins regulating cellular processes such as adhesion, cytoskeleton organization, cell permeability and therefore could be involved in the maintenance of BBB integrity. Despite these results needing to be confirmed *in vivo*, this study explores the gut/brain axis in a new and original way: the action of metabolites from the gut microbiota on BBB function and unravels an unexplored until recently an original mechanism by which flavonoids could exert their well-known neuroprotective effects.

*Grewia asiatica*, a popular native berry of Pakistan represents a very promising plant to treat and/or prevent various pathologies linked to oxidative stress and inflammation. The authors of the study showed that the berry contains various (poly)phenols including phenolic compounds, anthocyanins and flavonoids. Following 28 days of *Grewia asiatica* consumption in drinking water, the rats were subjected to diverse behavioral tests well-recognized to detect, quantify and analyze depression, anxiety, learning and memory. Interestingly, the rats treated with the highest doses of *Grewia asiatica* displayed low anxiety levels, similar to diazepam-treated animals, low depressive behavior similar to fluoxetine-treated animals and better learning and memory scores, counteracting the deleterious effect of scopolamine administration. Biochemical analysis of rat brains revealed that consumption of *Grewia asiatica* resulted in higher oxidative stress resistance indicated by elevated levels of superoxide dismutase and glutathione peroxidase and a reduction of malondialdehyde production. This effect was further paralleled by a beneficial modulation of the cholinergic system as indicated by reduced levels of acetylcholinesterase (Imran et al.).

The methanolic extract of *Otostegia Limbata*, a Lamiaceae plant, is commonly used as an active compound to potentially treat epilepsy. As many other (poly)phenol rich plants, *O. Limbata* demonstrated antioxidant (free-radical and NO scavenging and iron-chelating activities) andanti-inflammatory properties (reduced expression of P-NFkB and P-TNFa in the brain) potentially responsible for the anti-convulsant effect observed in mice model of epilepsy (Amin et al.).

We previously highlighted the necessity to study all the metabolites of each food-derived (poly)phenol and their by-product from microbiota, within various biological models, to better characterize the molecular action of these compounds in physiological conditions. And more specifically, when focusing on the brain, it's important to be able to answer the following questions: 1/ Which metabolite and at what dose does it cross the BBB 2/ Is the metabolite transformed during the uptake process 3/ Can brain cells metabolize (poly)phenolic compounds?

In conclusion, we need to investigate the specific molecular mechanisms for each (poly)phenol, their derivates and how they could potentially be metabolized in endothelial cells and diverse brain cells (neurons, astrocyte, tanycytes, and microglial cells), and then what would be the action of the resulting metabolites? Importantly the BBB isn't the only site of access to the brain for circulating molecules, circumventricular organs (CVO), highly vascularized structures located around the third and fourth ventricles and characterized by the lack of a classic BBB architecture, deserved to be studied along with the tanycytes, the main cellular component of the modified barrier around the CVOs (Langlet et al., 2013). These structures may also be involved in (poly)phenols transport and catabolism and could represent an interesting target to access the brain.

## Author contributions

EB wrote the manuscript. EB, DV, and PL edited the manuscript. All authors contributed to the article and approved the submitted version.

## Conflict of interest

The authors declare that the research was conducted in the absence of any commercial or financial relationships that could be construed as a potential conflict of interest.

## Publisher's note

All claims expressed in this article are solely those of the authors and do not necessarily represent those of their affiliated organizations, or those of the publisher, the editors and the reviewers. Any product that may be evaluated in this article, or claim that may be made by its manufacturer, is not guaranteed or endorsed by the publisher.
